# Investigation of the Effects of Different Home Frying Methods (Air Fryer and Deep Fryer) on HMF and Aroma Compounds in Gluten‐Free Bean Chips

**DOI:** 10.1002/fsn3.71065

**Published:** 2025-10-10

**Authors:** Ali Göncü

**Affiliations:** ^1^ Food Processing Department, Çine Vocational School Aydın Adnan Menderes University Aydın Türkiye

**Keywords:** air fryer, black beans, flavor, gluten‐free, HMF, red beans

## Abstract

The aim of this study was to produce functional gluten‐free chips with red and black bean flour substitutes using different household frying devices (deep fat fryer and hot air fryer) and to determine the flavor components and 5‐hydroxymethylfurfural (HMF) released during the production of these chips. The physicochemical, bioactive, and toxicological properties of the flours used in the study and the gluten‐free chips produced, as well as the flavor profile, and textural and sensory properties of the gluten‐free chips, were also determined. The study results are presented in a comparative manner for both frying methods and the correlation of HMF with other parameters. According to the results, chips produced with air fryer technology had much lower fat content and lighter color values (*L*: 67.04 and Browning Index (BI): 7.99). Conversely, they were harder (hardness of 8187.8 g) and had a higher HMF content (7.54 ppm). When we looked at the correlations for HMF, the strongest correlations were found between pH and *b* color values. In total, 67 flavor components were identified. The main volatile compounds of the chips were Benzene, 1‐methyl‐3‐(1‐methylethyl)‐, Benzene, 1,3‐dimethyl‐, .beta.‐Myrcene, .gamma.‐Terpinene, o‐Cymene, p‐Cymene, o‐Xylene, p‐Xylene, Acetic acid‐trichloro‐, methyl ester, Acetic acid‐trichloro‐anhydride, 18,18′‐Bi‐1,4,7,10,13,16‐hexaoxacyclononadecane, and 1,4,7,10,13,16‐Hexaoxacyclooctadecane. From a sensory evaluation, the most highly rated chips were those prepared with a 20% red bean flour substitution and fried in an air fryer. The results showed that the air fryer‐produced chips had lower fat content but were riskier in terms of HMF and were less appreciated from a sensory point of view.

## Graphical abstract

In this study, functional gluten‐free chips were produced using red and black bean flours and different frying methods (deep fat and hot air frying), and HMF and flavor components were analyzed. Chips fried with hot air had lower fat content but were harder, lighter in color, and had higher HMF levels. Sensory evaluation showed that the most preferred chips were those with 20% red bean flour, prepared using a hot air fryer.

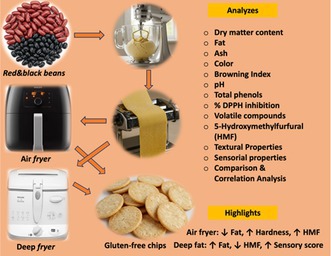

## 1 Introduction

Wheat flour contains gluten protein, which can have an adverse effect when consumed by people with celiac disease, gluten ataxia, and non‐celiac gluten sensitivity (Marta et al. 2024). These effects cause intestinal (diarrhea, bloating, and constipation) and extra‐intestinal (weight loss, anemia, osteoporosis, dermatitis herpetiformis, and neurological disorders) symptoms (Mazzola et al. 2024). Gluten‐related disorders can be classified into three categories according to their pathogenesis: autoimmune (celiac disease), allergic (IgE‐ or non‐IgE‐mediated wheat allergy), and autoimmune/non‐allergic (non‐celiac gluten sensitivity) (Sabença et al. 2021).

With the increasing number of celiac patients, the need for gluten‐free products is increasing (Mahmoud et al. 2013). Some people also choose to avoid gluten as part of their lifestyle. In response to the growing market demand for such products, the production of gluten‐free products has increased (Marta et al. 2024). Commercially available gluten‐free products are of low quality. These products, whose composition is based on starch, stale quickly and have negative properties due to the lack of nutrient content (Moroni et al. 2009). In the production of gluten‐free products, gluten‐free cereal and legume flours or starches such as rice, corn, soybeans, peanuts, chickpeas, and flours of cereal‐like products such as buckwheat, quinoa, and amaranth are used (Hayıt and Gül 2017). Hydrocolloids such as guar gum are also often used in gluten‐free products because some rheological properties of gluten are mimicked to improve the baking quality of gluten‐free flour. They also play a role in the texture, appearance, and stability of the product (Kalani et al. 2024). The addition of legume flours to gluten‐free foods is of interest because they can increase the protein biological value of the flour blend by complementing the properties of cereal flour, especially in terms of their amino acid profile. Moreover, they lower the glycemic index (GI) of the food products in which they are present (Wesley et al. 2021).

Legumes contain 18%–35% protein and are a source of fiber (resistant starch and dietary fiber), minerals, vitamins, amino acids, polyphenols, and bioactive peptides. They are poor in fat and calories (Lozano‐Aguirre et al. 2023; Galgano et al. 2023). Beans ( *Phaseolus vulgaris* L.) are one of the most important legumes, with 14%–33% protein content, amino acids such as lysine and phenylalanine, and tyrosine (Lozano‐Aguirre et al. 2023). Red beans, also known as adzuki beans and legume beans, are an annual plant. The protein content of red beans is about 20%, with one‐third of the amino acids being essential amino acids. As a high‐quality dietary protein, the digestion and absorption rate of red bean protein is high, up to more than 50% (Li et al. 2024). Black beans are also an excellent source of protein, dietary fiber, vitamins, and bioactive compounds such as isoflavones and phenolic acids (Guo 2024) and are considered to provide health benefits as functional foods with a low glycemic index (Gu et al. 2024).

Kayacier et al. (2014) reported that legume flour can be effectively used in wheat chip formulation to enhance the nutritional value in Europe and worldwide. Different heat treatments applied during the production of chips, such as cooking and frying, while improving the sensory properties of these products, also lead to the formation of some undesirable heat treatment contaminants (Basaran and Sadighara 2024). The health risks associated with a diet rich in fat are well known, and consumers are increasingly aware of the high fat content of fried foods, which have lost some of their positive nutritional value due to thermal damage. In this context, several companies have proposed many alternative devices aimed at replacing deep‐frying with cooking systems that can achieve similar sensory characteristics with better nutritional quality and ease of use (Giovanelli et al. 2017). The rapid airflow technology (Air fryer), which has recently become widespread, can be used to cook a variety of foods. This technology works by circulating superheated air in the cooking chamber, allowing for a more even distribution of temperature within the oven chamber. Therefore, the temperature setting can be adjusted to produce food of similar quality (Mior Zakuan Azmi et al. 2019). Some end products of the Maillard reaction can be toxic and carcinogenic and occur especially in baked or fried products (French fries) (Yakıcı 2012).

The Maillard reaction, another important flavor formation reaction involving lipids, takes place in heat‐treated foods. This process results in the formation of large amounts of volatile compounds as a result of thermal degradation, especially aldehydes, alcohols, furans, and ketones formed by the oxidation of alkyl chains of fatty acids (Ho and Chen 1994; Taylord and Linforth 2010). The flavor of chips is influenced not only by the type of raw material but also by the composition of the frying oil, temperature, and frying time (Martin and Ames 2001a, 2001b). While raw potatoes have little aroma, more than 140 volatile compounds have been identified in boiled potatoes, more than 250 in baked potatoes, and more than 500 in French fries. Among these, many lipid oxidation and Maillard reaction products have been reported, with smaller amounts of local flavor compounds (Comandini et al. 2011). An important factor influencing consumer preferences for fried food is its flavor, defined as the combined perception of aroma, taste, and mouthfeel (Montaser et al. 2017).

5‐hydroxymethylfurfural (HMF) is a toxic substance that occurs in certain foods during thermal processing using common high‐temperature unit operations such as frying, baking, and extrusion. Understanding the formation pathways of these potential risk factors, which can cause cancer or contribute to the development of many chronic diseases in humans, is crucial to reducing their occurrence in thermally processed foods. During thermal processing, foods rich in carbohydrates, proteins, and lipids undergo an important Maillard reaction leading to the production of highly active carbonyl compounds (Xiong et al. 2024; Zhang et al. 2024). These toxic compounds are primarily found in fried chips and baked bread products and have been linked to the development of cancer and chronic diseases in humans (Pedreschi et al. 2021).

The aim of this study was to increase the functionality of chips prepared from gluten‐free flours substituted with bean flours using different home frying systems and to detect both the aroma and color formation and the toxic compound HMF formed by the Maillard reaction during heat treatment. It is also to provide a comparative evaluation of the quality characteristics (physicochemical, bioactive, textural, and sensory) of the chips. Gluten‐free chips were prepared using deep fat fryers and air fryers. No study on gluten‐free chips has been found on this subject.

## 2 Materials and Methods

### 2.1 Materials

Gluten‐free black bean, red bean, corn, rice, and potato flours, corn starch, and guar gum were obtained from a gluten‐free raw material vendor (Ingro Gıda, İstanbul), while corn oil (Hüner Yağ, Adana) and salt (Billur Tuz, İzmir) were obtained from a local market in Aydın (Türkiye).

### 2.2 Methods

#### 2.2.1 Production of Chips

Chips were produced by slightly modifying the method of Kaplan et al. (2021). The ingredients given in Table 1, except water and oil, were mixed for 5 min to homogenize. To the homogenized gluten‐free flour mix, 50 ± 5 mL of drinking water and 1.5 g of corn oil for the ones to be fried in air fry were added, and the dough was obtained by kneading on the 2nd setting of the mixer (Kiwi Mixer, KMX‐3635, Türkiye) for 5 min. The dough was wrapped in cling film and rested for 30 min to ensure the required hydration. The rested dough was rolled out to a thickness of 1 mm using the 6th setting of the dough rolling apparatus (Tuğra Çelik, P247895s8441, Türkiye) and cut with a 5 cm diameter mold and made ready for frying. The chips were fried in deep oil using a deep fryer (Philips Fryer, HD6151/80, The Netherlands) at 180°C for 70 s and in an air fryer (Philips Airfryer XXL, HD9650/90, The Netherlands) using the recipe given for potato chips in the mobile application (Philips NutriU app) at 165°C for 10 min. Finally, they were cooled at room temperature and analyzed in 3 parallels.

**TABLE 1:** Formulations of the chips.

Sample^a^	Red bean flour (g)	Black bean flour (g)	Corn flour (g)	Rice flour (g)	Corn starch (g)	Potato flour (g)	Guar gum (g)	Salt (g)	Water (mL)
AFC	0	0	45	30	15	6	2	2	50 ± 5
AR10	10	0	40	25	15	6	2	2	50 ± 5
AR20	20	0	35	20	15	6	2	2	50 ± 5
AR30	30	0	30	15	15	6	2	2	50 ± 5
AB10	0	10	40	25	15	6	2	2	50 ± 5
AB20	0	20	35	20	15	6	2	2	50 ± 5
AB30	0	30	30	15	15	6	2	2	50 ± 5
DFC	0	0	45	30	15	6	2	2	50 ± 5
DR10	10	0	40	25	15	6	2	2	50 ± 5
DR20	20	0	35	20	15	6	2	2	50 ± 5
DR30	30	0	30	15	15	6	2	2	50 ± 5
DB10	0	10	40	25	15	6	2	2	50 ± 5
DB20	0	20	35	20	15	6	2	2	50 ± 5
DB30	0	30	30	15	15	6	2	2	50 ± 5

Abbreviations: AB10, airfry produced 10% black bean substitute chips; AB20, airfry produced 20% black bean substitute chips; AB30, airfry produced 30% black bean substitute chips; ACF, airfry produced control chips; AR10, airfry produced chips with 10% red bean substitution; AR20, airfry produced chips with 20% red bean substitution; AR30, airfry produced chips with 30% red bean substitution; DB10, deep fat 10% black bean substitute chips; DB20, deep fat 20% black bean substitute chips; DB30, deep fat 30% black bean substitute chips; DFC, deep fat control chips; DR10, deep fat 10% red bean substitute chips; DR20, deep fat 20% red bean substitute chips; DR30, deep fat 30% red bean substitute chips.

^a^ Bean flours were substituted for corn‐rice flours in the proportions indicated.

#### 2.2.2 Analyzes Physicochemical Analysis

Dry matter (method 934.01), ash (method 942.05), and fat (method 954.02) contents of the chips were determined according to AOAC (2005). Color values (*L*, *a*, and *b*) were determined using Hunter‐Lab Mini Scan XE colorimeter (Reston, VA, USA) (HunterLab 1995). Using the *L*, *a*, and *b* color values, the total color change (∆*E*) relative to the control chip samples was calculated using Equation (1) below.
(1)

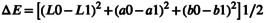




In the given equation, *L*0, *a*0, and *b*0 represent the color values of the control sample; *L*1, *a*1, and *b*1 represent the color values of the samples compared with the control (Göncü 2024). Browning Index (BI) values were calculated using Equation (2) below (Ding and Ling 2014):

(2) $$\mathrm{BI}=\left[100\;\left(x-0.31\right)\right]/0.17,\text{where}\;x=\left(a+1.75L\right)/\left(5.645L+a-0.3012b\right)$$

To determine the textural properties of gluten‐free chips, hardness values were determined using a texture analyzer (TA.XT2 Texture Analyzer). A P/2 2 mm cylindrical probe was used in the analysis and the pre‐test speed was set as 2 mm/s, the test speed as 2 mm/s, and the post‐test speed as 10 mm/s. The trigger force was set to 30 g (Yang et al. 2019).

#### 2.2.3 Bioactive Properties

Extracts were prepared for total phenolic content and antioxidant activity analysis. First, 4 g of ground samples were kept in a shaking water bath for 2 h at room temperature with 20 mL of acidification solution containing HCl:methanol:water (1:80:10, v/v/v). After centrifugation at 3000 rpm for 10 min, the supernatant was used for analysis (Yıldırım Vardin 2024).

The total phenolic content of the samples was determined spectrophotometrically. For this purpose, 0.5 mL of 10% Folin Ciocalteau reagent (v/v, in water) and 1.5 mL of 20% sodium carbonate (v/v, in water) solutions were added to 0.1 mL of the sample, and the samples were kept at room temperature and in the dark for 2 h. Then the absorbance was measured at 760 nm wavelength in a spectrophotometer (Thermo Scientific Multiskan GO spectrophotometer, ThermoFisher Scientific). The results were expressed as mg GAE/g (Karakoç 2021; Gamez‐Meza et al. 1999). For antioxidant activity measurement, DPPH stock solution and DPPH working solutions were prepared first. Then, 2850 μL of DPPH working solution was added to 150 μL of sample extract. Then the sample was mixed homogeneously and kept at room temperature in the dark for 1 h. The absorbance was then measured in a spectrophotometer (Thermo Scientific Multiskan GO spectrophotometer, ThermoFisher Scientific, United States of America) at a wavelength of 515 nm. The results were expressed as μmol TE/100 g (Yıldırım Vardin 2024).

#### 2.2.4 HMF Analysis

To determine HMF, 6.9 g of sample was taken and diluted to 50 mL with distilled water. The samples were then filtered through a 0.45‐μm membrane filter to remove impurities. The samples were then injected into an HPLC device (HPLC, Agilent Technologies, California/USA). The amount of HMF in the samples was quantitatively determined using the calibration curve prepared with different concentrations of HMF standard. The mobile phase was prepared with 80% distilled water and 20% methanol at a flow rate of 1 mL/min. Chromatographic separation was carried out at room temperature at a wavelength of 285 nm in a DAD detector. Characteristics of the column used: C18, particle diameter 3 μm, column *L* × ID; 150 × 4.6 (Göncü 2024).

#### 2.2.5 Aroma Analysis

The volatile components of the samples were analyzed by gas chromatography–mass spectrometry (GC–MS) employing the solid‐phase micro‐extraction (SPME) technique (D'Auria et al. 2004). Firstly, 2 g of the sample was weighed into vials with septum caps and incubated at 40°C for 30 min to facilitate the transfer of aroma compounds from the sample to the surrounding medium. A DVB/CAR/PDMS fiber (65 μm, Supelco, Bellefonte PA/USA) was introduced into the headspace of the vial for SPME and maintained at 40°C for an additional 30 min. Subsequently, the fiber‐containing volatile components were transferred to the GC–MS sampling port. The analysis of volatile components was performed using a capillary column (Restek Rxi‐5 ms, USA; 30 m × 0.25 mm ID × 0.25 μm). The injection port temperature was 250°C, the interface temperature was 250°C, and the desorption time was 3 min. High‐purity helium was the mobile phase. Injection was done in splitless mode. GC‐column temperature was as follows: Starting with 1 min at 50°C, followed by a gradual increase of 3°C/min to 200°C, then 8°C/min to 250°C, with a hold at 250°C for 5 min; MS‐scan mode was set to 35–450 m/z; ionization energy was at 70 eV. Identification of volatile compounds was carried out using libraries registered on GC–MS.

#### 2.2.6 Sensory Analysis

The sensory analysis was carried out by 58 researchers aged between 18 and 44 years (15 men and 43 women) and semi‐trained in sensory evaluation. Gluten‐free chips were coded with three‐digit random numbers. Samples were served to panelists on white plastic plates at individual tables in a randomized order. Panelists were given unsalted bread and water to neutralize and rinse their mouths before testing each sample. Panelists were asked to provide hedonic ratings on seven‐point structured scales (1 = extreme dislike; 7 = extreme liking) for taste, smell, color, crunchiness, and overall liking. According to the ethical guidelines of Aydın Adnan Menderes University, ethics committee approval was not required for this study. All participants were informed about the study and provided their consent before participation.

### 2.3 Statistical Analysis

The data were analyzed using “Minitab 16 Statistical Program”. ANOVA (one‐way analysis of variance) and Tukey (multiple comparison test) tests were used to detect significant differences (*α* = 0.05). In addition, Pearson correlation analysis was performed between the data in order to better understand the formation of heat treatment contaminants.

## 3 Results and Discussion

Some properties of the flours used in gluten‐free chip production are given in Table 2. When the data were analyzed, the values were determined in the ranges of dry matter: 96.29%–99.80%; ash: 0.99%–3.74%; fat: 0.36%–4.08%; *L*: 83.19–90.16; *a*: −0.21–3.05; and *b*: 7.34–25.39; ranges were determined. Due to the high dietary fiber content (Le et al. 2024), the ash content of both red and black bean flours was higher than that of the other flours. Corn flour was found to be the richest in fat while potato flour was the lowest. In a study (Campos‐Vega et al. 2009), the % fat and % ash contents of different beans were given as follows: 0.8–2.0 and 3.7–4.7. While the fat values of the bean flours we used in the study were within these limits, in terms of ash, black bean flour was within the limits while red bean flour was slightly below. In another study (Barreto et al. 2021), the % fat and % ash amounts of black bean flours grown in Brazil under different organic and normal conditions were given in the following ranges: 1.4–19 and 3.8–4.4. When the color characteristics were examined, the *L* value was found to be the highest in rice flour with a white color, while black bean flour had the lowest *L* value due to the dark pigments it contains. When *a* and *b* values were considered, corn flour gave the highest redness and yellowness values. In a study (de la Hera et al. 2013), the color values of corn flours with different particle sizes were given as follows: *L*: 87.66–89.93; *a*: 4.21–5.65; and *b*: 33.60–38.52. Although the *L* value of the corn flour we used in the production of chips was within the given range, the *a* and *b* values were lower. If we compare white bean flour with red and black bean flour, it is understood that white bean flour has much lower dry matter, ash, and fat content (88.67%, 1.92%, and 1.10%, respectively) (Wesley et al. 2021). Chávez‐Mendoza et al. (2018) gave the color values of 13 different bean species in the following range: *L*: 23.47–77.00; *a*: 0.78–15.46; and *b*: −1.82–33.31. It can be said that the reason for these different results found for flours is due to different species and cultivation under different seasonal conditions.

**TABLE 2:** Physicochemical properties of flours (%)*.

Sample*	Dry matter (%)**	Ash (%)**	Oil (%)**	*L*	*a*	*b*
Red bean flour	97.72 ± 0.02b	3.47 ± 0.35a	1.24 ± 0.01c	83.19 ± 0.01d	1.59 ± 0.01b	8.58 ± 0.01d
Black bean flour	97.25 ± 0.12b	3.74 ± 0.25a	2.00 ± 0.08b	78.16 ± 0.02e	1.12 ± 0.01c	7.34 ± 0.01e
Corn flour	96.39 ± 0.22c	1.13 ± 0.19c	4.08 ± 0.15a	87.84 ± 0.01b	3.05 ± 0.01a	25.39 ± 0.01a
Rice flour	96.29 ± 0.36c	0.99 ± 0.14c	1.20 ± 0.01c	90.16 ± 0.01a	0.10 ± 0.01d	11.09 ± 0.02c
Potato flour	99.80 ± 0.08a	2.46 ± 0.03b	0.36 ± 0.03d	87.40 ± 0.03c	−0.21 ± 0.01e	18.43 ± 0.03b

* Different letters in the same column indicate that the results are statistically different (*p* < 0.05).

** Results are given according to dry matter.

Table 3 shows the bioactive and toxicological properties of gluten‐free flours. The pH, total phenolic content, antioxidant activity, and HMF content of the flours were found in the following ranges respectively: 6.23–7.18, 0.17–0.99, 0.16–0.22, and 0–10.43. Red bean flour and corn flour were the richest in terms of total phenolic content. Corn grain contains a wide range of phytonutrients, including antioxidants. It was reported by Žilić et al. (2010) that it contains high levels of antioxidant compounds such as carotenoids, tocopherols, and phenolics. Similarly, it is estimated to contain high amounts of total phenolic substances in our study. It has also been emphasized by researchers (Sancho et al. 2015; Le et al. 2024) that red bean flour, which has the highest value, followed by black bean flour, has a rich phenolic content and these are due to the presence of anthocyanins, flavonoids, phenolic acids, flavon‐3‐ols, and tannins. Although the amounts of total phenolic substances were different, no significant change was found in the antioxidant values of the flours. In the study conducted by Sancho et al. (2015), there was no statistical difference in the analysis results of red and black beans determined by some methods. Carbas et al. (2020) found that the total phenolic content of black beans (2.25 mg GAE/g) was lower than that of red beans (2.70–4.59 mg GAE/g). In the same study, although the antioxidant activity results were in parallel with the total phenolic content, no significant difference was found in our study. In another study (Chávez‐Mendoza et al. 2018), some bean varieties grown in Mexico were examined and total phenolic matter amounts were given as 0.99–3.32 mg GAE/g. While the total phenolic content of red bean flour used in the production of chips was in this range, black bean flour was found to be lower. These differences are thought to be due to raw materials or methods. When the toxicological properties of the flours were analyzed, it was found that HMF was found only in bean flours, and it was too low to be detected in other flours. Similarly, Mesías and Morales (2017) reported that HMF amounts in cereal and pseudocereal flours such as wheat, dinkel (spelt), oat, teff, and rye were below the detection limit.

**TABLE 3:** Bioactive and toxicological properties of flours**.

Sample*	pH	Total phenolic substance (mg GAE/g)	Antioxidant activity (μmol TE/100 g)	HMF (ppm)
Red bean flour	6.72 ± 0.01b	0.99 ± 0.13a	0.22 ± 0.02a	7.68 ± 0.09
Black bean flour	6.55 ± 0.02c	0.63 ± 0.09bc	0.16 ± 0.06a	10.43 ± 0.24
Corn flour	6.45 ± 0.02d	0.75 ± 0.11ab	0.18 ± 0.02a	<LOQ
Rice flour	7.18 ± 0.03a	0.48 ± 0.03c	0.19 ± 0.01a	<LOQ
Potato flour	6.23 ± 0.05e	0.17 ± 0.01d	0.20 ± 0.20a	<LOQ

Abbreviation: LOQ, limit of quantification.

* Different letters in the same column indicate that the results are statistically different (*p* < 0.05).

** Results are given according to dry matter.

Some physicochemical properties of the chips are given in Table 4. The dry matter, ash, and fat contents (%) of gluten‐free chips were found in the ranges of 96.96–99.80, 2.62–4.58, and 2.92–24.23, respectively. Dry matter content of wheat chips prepared with chickpea, pea, and soybean flours: 96.42%–99.79%; ash: 0.64%–1.42%; and fat: 18.69%–35.44% (Kayacier et al. 2014). In a study, gluten‐free chips were obtained with a mix of millet flour, adzuki bean flour, and basil seed flour and the dry matter content was 93.79%; ash: 3.9%; and fat: 9.0% (Agarwal and Chauhan 2022). The dry matter content of sorghum‐substituted gluten‐free chips obtained by deep‐frying was: 99.22%; ash: 2.13%; and fat: 29.01% (Kaplan et al. 2021). The dry matter content of gluten‐free chips produced by cooking and substituting almond flour was obtained by different processes: 93.0%–97.84% and fat 1.84%–9.11% (Altay et al. 2023). In the study of Yüksel et al. (2019), the dry matter content of gluten‐free chips obtained by deep‐frying after pre‐drying: 94.55%–99.12%; ash 2.06%–2.97%; fat: 13.06%–23.07%. The fat content of chips with gluten, bran, germ, and whey powder addition produced by cooking between hot toast plates used in wafer production was reported to be a maximum of 2.50% (Ertop et al. 2016). The dry matter and fat contents of gluten‐free chips deep‐fried in oil were measured higher than those dried in a hot air fryer. This is consistent with the studies given above. Ash content was found to be consistent with some studies and different from some studies. These differences are due to the different formulations used in the studies. Since a high amount of advanced glycation end products are formed in processed cereal products, especially when they are prepared in oil, and pH is reported to cause changes in the variety and amounts of contaminants that are Maillard products (Cengiz et al. 2020), pH measurements were made to determine the effects on HMF.

**TABLE 4:** Some physicochemical properties of chips**.

Sample*	Dry matter (%)	Ash (%)	Oil (%)	pH	Hardness (g)
AFC	99.27 ± 0.26ab	2.92 ± 0.38def	2.97 ± 0.06e	6.44 ± 0.02a	8620.0 ± 17.5a
AR10	98.42 ± 0.14bc	3.66 ± 0.06abcde	2.96 ± 0.09e	6.27 ± 0.02b	8330.6 ± 15.4b
AR20	97.45 ± 0.12 cd	3.97 ± 0.20abc	2.89 ± 0.05e	6.26 ± 0.03b	8112.6 ± 18.9d
AR30	96.96 ± 0.49d	3.65 ± 0.05abcde	2.92 ± 0.01e	6.25 ± 0.01b	7948.0 ± 15.8e
AB10	97.40 ± 0.54 cd	3.53 ± 0.16bcdef	3.15 ± 0.18e	6.15 ± 0.01c	8218.4 ± 20.6c
AB20	97.20 ± 0.57 cd	4.13 ± 0.02ab	3.06 ± 0.20e	6.08 ± 0.01c	8105.1 ± 9.2d
AB30	98.51 ± 0.23bc	4.58 ± 0.11a	2.94 ± 0.02e	6.14 ± 0.01c	7980.0 ± 14.1e
DFC	98.81 ± 0.71ab	2.62 ± 0.13f	21.16 ± 0.81d	6.43 ± 0.06a	7609.6 ± 17.6f
DR10	99.40 ± 0.25ab	2.62 ± 0.38f	21.11 ± 0.64d	6.40 ± 0.01a	7513.7 ± 25.1 g
DR20	99.49 ± 0.37ab	3.85 ± 0.22abcd	22.49 ± 0.49c	6.40 ± 0.02a	7215.7 ± 36.0i
DR30	98.96 ± 0.25ab	2.78 ± 0.26ef	23.63 ± 0.33ab	6.39 ± 0.02a	7209.6 ± 11.6i
DB10	99.77 ± 0.11a	2.90 ± 0.58def	22.71 ± 0.33bc	6.26 ± 0.01b	7326.8 ± 18.4 h
DB20	98.41 ± 0.49bc	3.04 ± 0.36cdef	24.23 ± 0.33a	6.26 ± 0.02b	7190.1 ± 14.4i
DB30	99.80 ± 0.09a	3.70 ± 0.73abcde	23.02 ± 0.17bc	7096.6 ± 8.3j	7096.6 ± 8.3j

* Different letters in the same column indicate that the results are statistically different (*p* < 0.05).

** Results are given according to dry matter.

The pH value of red bean flour was higher than that of black bean flour (Table 3). This result was also observed in the chips. Chips made with red bean flour had higher pH values than chips made with black bean flour, both in an air fryer and when deep fried. Table 7 shows the correlation between pH and HMF.

Yüksel (2014) stated that the hardness of chips is important because chips should be durable enough not to crack until they reach the consumer. Yuksel and Kayacier (2016) reported that chips should be hard enough to withstand cracking during processing and in the packaging until they reach the consumer and should be crispy when brought to the mouth to chew. In a study (Kaplan et al. 2021), it was reported that hardness decreased with increasing sorghum substitution in gluten‐free chips. In addition, hardness levels were expressed as 9.68–10.84 kg. The hardness values of corn chips enriched with stale bread powder were reported to be in the range of 10.64–25.67 kg, and it was reported that the hardness properties of the chips were affected by the addition of stale bread powder (Yuksel et al. 2017). In the study of Altay et al. (2023), the increase in the addition of almond flour caused a decrease in the hardness of gluten‐free chips. In another study (Kayacier et al. 2014), different legume flours were substituted for wheat flour, and deep‐fried and hardness levels were reported in the range of 15950.2–26371.0 g. When compared with our study, it is understood that these values are higher. It is thought that the reason for this is that chips produced with different raw materials were analyzed with different probes. Lisińska and Gołubowska (2005), who investigated the effects of frying conditions and moisture value on potato chips, reported that increasing the frying temperature caused a significant decrease in chip hardness. Yi et al. (2015) also found that the hardness value of chips obtained by deep frying was lower than those produced in the microwave. In parallel with the studies, the hardness values of the chips decreased as the substitution of bean flours increased and the deep‐frying process was carried out at higher temperatures. Olegario et al. (2023) reported that reducing the fat in potato chips significantly improved the texture sensations. This is consistent with our results. It was determined that the chips were harder with airfry application where the amount of oil was less.

The color values of the chips are given in Table 5. Since red and black bean flours were darker in color than the gluten‐free flour mix from which we produced the control chips, the *L* values of the chips substituted with bean flours were lower. This decrease became more significant as the substitution rates increased. The *L* values of the deep‐fried chips were lower than those fried in a hot air fryer. When the values of the chips were analyzed, it was observed that they increased with the substitution of red bean flour. The *b* value decreased as the substitution rates increased and was higher in deep‐fried chips. These results were also supported by ∆*E* values. The ∆*E* value increased as the substitution rate increased and black bean flour was used.

**TABLE 5:** Color values of chips.

Sample*	*L*	*a*	*b*	∆*E*	BI
AFC	78.80 ± 0.48a	4.81 ± 0.36hi	25.56 ± 0.68c	—	7.60 ± 0.46hi
AR10	74.08 ± 0.14b	5.17 ± 0.17 gh	22.69 ± 0.44d	5.55 ± 0.45 g	8.03 ± 0.20hi
AR20	71.73 ± 0.22c	5.75 ± 0.06 fg	21.61 ± 0.15d	8.18 ± 0.23f	8.72 ± 0.06gh
AR30	69.39 ± 0.83d	6.46 ± 0.22f	21.56 ± 0.38d	10.37 ± 0.74e	9.72 ± 0.17 g
AB10	65.98 ± 0.64e	4.09 ± 0.05ij	17.91 ± 0.45e	14.96 ± 0.66d	7.13 ± 0.06i
AB20	58.91 ± 1.11 g	3.89 ± 0.39j	13.37 ± 0.51f	23.39 ± 0.87b	6.97 ± 0.69i
AB30	50.42 ± 0.30i	3.58 ± 0.13j	13.58 ± 0.38f	30.84 ± 0.24a	7.75 ± 0.18hi
DFC	63.84 ± 0.06f	7.73 ± 0.08e	32.63 ± 0.01a	—	13.76 ± 0.08f
DR10	60.01 ± 0.64 g	12.02 ± 0.40c	32.28 ± 0.41a	5.77 ± 0.64 g	19.51 ± 0.57c
DR20	54.84 ± 0.48 h	13.20 ± 0.22b	31.54 ± 0.79a	10.61 ± 0.39e	22.64 ± 0.23b
DR30	51.66 ± 0.10i	14.69 ± 0.30a	30.92 ± 0.84a	14.14 ± 0.27d	25.79 ± 0.43a
DB10	54.52 ± 0.84 h	8.75 ± 0.16d	27.90 ± 0.89b	10.53 ± 0.80e	16.50 ± 0.35de
DB20	50.02 ± 0.85i	7.69 ± 0.56e	23.17 ± 0.98d	16.78 ± 0.18c	15.54 ± 1.22e
DB30	43.65 ± 0.44j	8.14 ± 0.39de	21.37 ± 1.13d	23.13 ± 0.73b	18.05 ± 0.91cd

* Different letters in the same column indicate that the results are statistically different (*p* < 0.05).

Rababah et al. (2012) reported that the redness values of chips samples increased with the increase in frying temperature because of the interaction between the Maillard reaction and frying temperature. Qadri et al. (2018) similarly reported that Maillard and caramelization reactions cause color change in products at high temperatures. Yuksel (2017) also stated that since the Maillard reaction occurs at high temperatures, the color values of chips are affected by the Maillard reaction. This explains why the *L* values of chips fried in deep oil at higher temperatures are lower, but the *a* and *b* values are higher. Yüksel et al. (2019) explained the color values of gluten‐free chips produced by applying pre‐heat treatment in the following ranges: *L*: 43.14–63.19; *a*: 4.64–13.56; and *b*: 15.69–28.63. Kaplan et al. (2021) reported the color values of gluten‐free chips as *L*: 64.90; *a*: 4.31 and *b*: 34.70. The color values of our results and similar products in the literature were found to be close to each other. BI was detected more in deep‐fried gluten‐free chips because it is associated with color changes and more with *a* value. Yi et al. (2015) produced potato chips both in deep fat and in a microwave oven and found that the BI value was higher in deep fat. In general, color changes are like our study.

The bioactive and toxicological properties of the chips are given in Table 6. The total phenolic content and antioxidant activity values of gluten‐free chips ranged from 0.16 to 0.46 mg GAE/100 g and 0.16 to 0.29 μmol TE/100 g, which are lower than those reported by Göncü and Hayta (2018) for baked wheat chips, Rocchetti et al. (2018) for corn snack with red sorghum flour, and Halil et al. (2020) for chickpea and bean chips with green olives (Table 6). These differences may be related to differences in the process and the variety of raw materials. It has been reported that HMF and acrylamide formation are positively correlated in potato chip production, and the presence and amount of HMF is an indicator for acrylamide content (Pedreschi et al. 2021). From this point of view, the detection of HMF is very important in chip production. In addition, since it is one of the main intermediates of the Maillard reaction, it is a quality indicator because it is related to the severity of heat treatment (Ertekin Filiz and Seydim 2018). In this study, the HMF content of the chips was found between 6.02 and 8.99 ppm. The HMF content of chips produced in a hot air fryer was higher (*p* < 0.05) than those deep‐fried. Among the chips produced in hot air fryers, those with black bean flour substitutes had higher HMF content than those with red bean flour substitutes. This result is also consistent with the initial HMF contents of the raw materials. When the raw materials are analyzed, it is seen that black bean flour contains more HMF than red bean flour. Bean flours with increasing HMF content did not undergo any change in their substitutes (*p* > 0.05). In a study (Mesias et al. 2019), 40 different traditional and 14 different innovative cereal‐based chips and snack types were evaluated in terms of HMF, and the results were given in the range of 0.6 to 91.3 ppm. The averages ranged between 7.00 and 18.52 ppm, respectively. Félix‐Medina et al. (2024) determined HMF amounts in the range of 0.9 to 1.8 ppm in snacks obtained with corn and beans in their study. These values are much lower than what we detected in gluten‐free chips. The fact that the results vary so much is related to the types and amounts of raw materials and the methods of obtaining the chips. While all the results we obtained in our study were well below the HMF values of the innovative formulations, those produced in deep oil and those obtained by a hot air fryer were found to be lower than those produced by the traditional method.

**TABLE 6:** Bioactive and toxicological properties of chips**.

Sample*	Total phenolic substance (mg GAE/g)	Antioxidant activity (μmol TE/100 g)	HMF (ppm)
AFC	0.39 ± 0.05abc	0.23 ± 0.01abc	7.20 ± 0.76bc
AR10	0.36 ± 0.03abcde	0.29 ± 0.06a	6.59 ± 0.72c
AR20	0.38 ± 0.03abcd	0.25 ± 0.01abc	6.42 ± 0.68c
AR30	0.37 ± 0.05abcde	0.25 ± 0.01abc	6.38 ± 0.62c
AB10	0.46 ± 0.05a	0.19 ± 0.04bc	8.99 ± 0.26a
AB20	0.44 ± 0.07ab	0.20 ± 0.03bc	8.85 ± 0.28a
AB30	0.43 ± 0.09abc	0.22 ± 0.01abc	8.33 ± 0.20ab
DFC	0.16 ± 0.01f	0.16 ± 0.01c	6.02 ± 0.01c
DR10	0.41 ± 0.03abc	0.26 ± 0.03ab	6.02 ± 0.01c
DR20	0.22 ± 0.01ef	0.19 ± 0.03bc	6.02 ± 0.01c
DR30	0.23 ± 0.07def	0.19 ± 0.02bc	6.43 ± 0.17c
DB10	0.19 ± 0.04f	0.19 ± 0.01bc	6.39 ± 0.15c
DB20	0.27 ± 0.05cdef	0.20 ± 0.01abc	6.85 ± 0.27c
DB30	0.28 ± 0.03bcdef	0.19 ± 0.02bc	6.87 ± 0.19c

* Different letters in the same column indicate that the results are statistically different (*p* < 0.05).

** Results are given according to dry matter.

The results of the Pearson correlation analysis performed to establish the possible relationship between heat treatment contaminants and other parameters are presented in Table 7. Significant correlations were observed between these toxic compounds formed in the production of gluten‐free chips and other parameters. There were positive correlations between HMF and ash, hardness and total phenolic content, and negative correlations with dry matter, fat, *a*, *b*, BI, and pH. There were strong correlations between pH and color values of *b* and HMF. Ertekin Filiz and Seydim (2018) stated that decreasing pH increases the reaction rate of HMF formation. In our study, in parallel with this, the HMF content of the chips obtained with a hot air fryer with a lower pH was found to be higher. In addition, as shown in Table 7, a very strong and negative correlation was found between pH and HMF. No correlation was found between *L* and antioxidant activity and HMF. The other correlations were statistically significant but not strongly correlated.

**TABLE 7:** Pearson correlation matrix (correlation coefficients (*r*) and *p* value).

	Dry matter	Ash	Oil	*L*	*a*	*b*
HMF	−0.423**	0.466**	−0.538***	NA	−0.638***	−0.786***

	∆*E*	BI	Hardness	pH	Total phenolic substance	Antioxidant activity
HMF	NA	−0.574***	0.442**	−0.714***	0.578***	NA

Abbreviation: NA, no correlation.

** *p* ≤ 0.01.

*** *p* ≤ 0.001.

Table 8 shows the relationship between a hot air fryer and a deep fat fryer. When the table is analyzed, all values except ∆*E* are statistically different. Since the deep‐frying process was carried out at a higher temperature, there was a significant difference in color values. While the *L* value decreased, the *a* and *b* values and BI increased. The hardness value decreased with the total phenolic content and antioxidant activity. However, HMF was lower. It is known that melanoidin formed in Maillard reactions has an antioxidative effect (Morales and Jiménez‐Pérez 2001), so it is thought that HMF and antioxidant activity are higher in chips made with an air fryer because the frying time in the air fryer is longer.

**TABLE 8:** Relationship between airfry and deep fat frying processes*.

	Dry matter (%)	Ash (%)	Oil (%)	*L*	*a*	*b*	HMF (ppm)
Airfry**	97.89 ± 0.87b	3.78 ± 0.51a	2.98 ± 0.12b	67.04 ± 9.17a	4.82 ± 1.01b	19.47 ± 4.44b	7.54 ± 1.19a
Fryer**	99.23 ± 0.59a	3.07 ± 0.59b	22.62 ± 1.18a	54.07 ± 6.29b	10.31 ± 2.78a	28.54 ± 4.40a	6.37 ± 0.38b

	∆*E*	BI	Hardness (g)	pH	Total Phenolic Substance (mg GAE/g)	Antioxidant Activity (μmol TE/100 g)
Airfry**	15.55 ± 9.18a	7.99 ± 0.95b	8187.8 ± 220.0a	6.23 ± 0.11b	0.40 ± 0.06a	0.23 ± 0.04a
Fryer**	13.49 ± 5.66a	18.83 ± 4.02a	7308.9 ± 178.5b	6.35 ± 0.07a	0.25 ± 0.08b	0.20 ± 0.03b

* Different letters in the same column indicate that the results are statistically different (*p* < 0.05).

** *N* = 21.

Table 9 shows the volatile compound profiles of the chips. In total, 67 different volatile compounds were identified. Major volatile compound profiles of chips Benzene, 1‐methyl‐3‐(1‐methylethyl)‐, Benzene, 1,3‐dimethyl‐, .beta.‐ Myrcene, .gamma.‐Terpinene, o‐Cymene, p‐Cymene, o‐Xylene, p‐Xylene, Acetic acid, trichloro‐, methyl ester, Acetic acid, trichloro‐, anhydride, 18,18′‐Bi‐1,4,7,10,13,16‐hexaoxacyclononadecane and 1,4,7,10,13,16‐hexaoxacyclooctadecane, 2‐[2‐[2‐[2‐[2‐[2‐[2‐[2‐[2‐(2Methoxyethoxy)ethoxy]ethoxy]ethoxy]ethoxy]ethoxy]ethoxy]ethoxy]ethoxy]ethanol, 2‐[2‐[2‐[2‐[2‐[2‐[2‐(2‐ Hydroxyethoxy)ethoxy]ethoxy]ethoxy]ethoxy]ethoxy]ethoxy]ethanol,′dir.

**TABLE 9:** Aroma component of chips.

Component (% area)	AFC	AR10	AR20	AR30	AB10	AB20	AB30	DFC	DR10	DR20	DR30	DB10	DB20	DB30
Benzene, 1,3‐dimethyl—	2.74	8.76	1.35	4.16	4.66	1.25	6.38	2.04	9.30	8.32	7.04	11.36	0.48	3.28
Benzene, 1‐methyl‐3‐(1‐methylethyl)—	0.00	0.00	15.22	0.00	0.00	0.00	6.98	0.00	0.00	0.00	16.98	0.00	17.41	6.02
Ethylbenzene	0.03	0.00	0.10	0.13	0.00	0.00	0.01	0.00	0.00	0.39	0.19	0.31	0.00	0.20
.beta.‐Myrcene	3.17	1.48	0.00	3.27	3.12	1.62	2.43	2.45	4.94	4.78	3.06	0.00	4.97	5.62
. gamma.‐Terpinene	12.29	7.00	10.22	11.62	10.80	11.38	7.87	10.43	9.51	10.71	8.38	7.24	8.39	8.05
(+)‐4‐Carene	0.00	0.00	0.00	1.70	0.00	0.00	0.61	0.76	2.40	2.16	0.00	0.00	2.33	0.00
o‐Cymene	16.97	0.00	15.78	17.31	15.80	18.20	0.00	15.35	15.44	15.99	0.00	6.88	0.00	0.00
p‐Cymene	0.00	15.52	0.00	19.80	16.90	15.24	17.04	17.76	13.87	0.00	11.00	0.00	0.00	18.06
o‐Xylene	4.12	5.10	0.79	0.00	0.00	1.18	2.91	0.05	7.07	3.30	4.68	4.88	8.38	4.75
p‐Xylene	3.09	2.20	5.43	3.82	5.51	2.00	3.85	6.75	2.78	4.33	1.93	0.00	5.02	7.74
2‐Heptenal, (E)—	0.02	0.27	0.08	0.00	0.00	0.36	0.08	0.00	0.00	0.00	0.00	0.00	0.00	0.00
Heptaethylene glycol	0.00	0.00	0.00	0.26	0.00	0.00	0.00	0.00	0.00	1.49	0.49	0.44	0.00	0.00
Acetic acid, trichloro‐, methyl ester	6.45	11.28	10.16	8.22	8.88	2.52	3.95	5.90	6.54	5.45	4.68	10.12	10.49	8.75
Acetic acid, trichloro‐, anhydride	9.85	15.01	13.96	13.71	13.86	6.00	6.93	7.67	7.24	6.52	6.36	10.25	8.06	6.45
Nonanal	0.00	0.00	0.66	0.00	0.00	0.00	0.00	0.31	0.21	0.26	0.00	0.00	0.16	0.17
Thujone	0.00	0.00	0.00	0.00	0.00	0.30	0.00	0.00	0.00	0.00	0.00	0.00	0.00	0.00
1‐Octanol	0.00	0.00	0.00	0.00	0.00	0.09	0.00	0.00	0.00	0.00	0.00	0.00	0.00	0.00
1‐Octen‐3‐ol	0.00	0.00	0.16	0.00	0.00	0.20	0.00	0.00	0.00	0.00	0.00	0.00	0.00	0.00
3‐Octen‐2‐one, (E)—	0.00	0.05	0.00	0.00	0.00	0.00	0.00	0.00	0.00	0.00	0.00	0.00	0.00	0.00
1,3‐Hexadiene, 3‐ethyl‐2‐methyl—	0.15	0.00	0.00	0.00	0.00	0.00	0.00	0.00	0.00	0.00	0.00	0.00	0.00	0.00
3,5‐Octadien‐2‐ol	0.00	0.07	0.08	0.00	0.00	0.13	0.00	0.00	0.00	0.00	0.00	0.00	0.00	0.00
2‐Octenal, (E)—	0.00	0.47	0.00	0.00	0.00	0.47	0.00	0.00	0.00	0.00	0.00	0.00	0.00	0.00
Benzene, 1,2‐dichloro—	0.14	0.00	0.27	0.20	0.18	0.00	0.00	0.00	0.00	0.00	0.00	0.00	0.00	0.00
Benzene, 1,3‐dichloro—	0.00	0.00	0.16	0.00	0.00	0.00	0.00	0.00	0.00	0.00	0.00	0.00	0.00	0.00
Benzene, 1,4‐dichloro—	0.00	0.00	0.15	0.00	0.00	0.00	0.00	0.00	0.00	0.00	0.00	0.00	0.00	0.00
Heptanoic acid	0.00	0.00	0.00	0.00	0.00	0.08	0.00	0.00	0.00	0.00	0.00	0.00	0.00	0.27
Furfural	0.54	1.06	1.01	1.39	1.25	1.27	1.61	0.00	0.00	0.00	0.00	0.00	0.00	0.00
2‐Ethyl‐1‐hexanol	0.00	0.29	0.00	0.00	0.00	0.00	0.14	0.00	0.00	0.00	0.00	0.00	0.00	0.00
1‐Hexanol, 2‐ethyl—	0.15	0.14	0.29	0.00	0.20	0.15	0.31	0.00	0.00	0.00	0.00	0.00	0.00	0.00
Tetracontane, 3,5,24‐trimethyl—	0.00	0.00	0.00	0.28	0.00	0.00	0.00	0.00	0.00	0.00	0.00	0.00	0.00	0.00
Benzaldehyde	0.83	1.14	1.27	1.06	1.14	0.00	0.78	0.00	0.00	0.87	0.00	0.00	0.00	0.00
3‐Hydroxymandelic acid, ethyl ester, di‐TMS	0.15	0.00	0.27	0.18	0.25	0.16	0.00	0.00	0.00	0.00	0.00	0.45	0.21	0.00
Phosphonoacetic Acid, 3TMS derivative	0.08	0.00	0.13	0.00	0.00	0.00	0.00	0.09	0.00	0.18	0.09	0.00	0.00	0.00
Acetophenone	0.19	0.00	0.00	0.14	0.00	0.14	0.00	0.00	0.00	0.00	0.00	0.00	0.00	0.00
Oxime‐, methoxy‐phenyl‐_	0.00	0.29	0.00	0.00	0.00	0.00	0.49	0.27	0.21	0.20	1.25	0.36	0.35	0.36
2,4‐Dihydroxybenzaldehyde, 2TMS derivative	0.00	0.00	0.28	0.00	0.18	0.00	0.00	0.00	0.00	0.00	0.00	0.23	0.00	0.22
Pentanoic acid	0.00	0.12	0.18	0.00	0.00	0.00	0.15	0.00	0.00	0.00	0.00	0.00	0.00	0.00
2,4‐Nonadienal, (E,E)—	0.00	0.00	0.00	0.00	0.00	0.13	0.00	0.00	0.00	0.00	0.00	0.00	0.00	0.00
2,4‐Decadienal, (E,E)—	0.12	0.22	0.28	0.00	0.22	0.64	0.22	0.68	0.52	1.07	0.59	0.71	0.70	0.67
Hexanoic acid	1.08	2.00	1.76	0.56	0.00	1.93	1.40	0.43	0.46	0.28	0.08	0.00	0.35	0.00
Benzyl alcohol	0.22	0.24	0.31	0.20	0.00	0.14	0.20	0.00	0.00	0.19	0.00	0.24	0.00	0.18
Phenylethyl Alcohol	0.33	0.00	0.00	0.14	0.00	0.14	0.00	0.00	0.00	0.00	0.00	0.00	0.00	0.00
2‐[2‐[2‐[2‐[2‐[2‐[2‐[2‐[2‐(2‐Methoxyethoxy)ethoxy]ethoxy]ethoxy]ethoxy] ethoxy]ethoxy]ethoxy]ethoxy]ethanol	9.91	5.33	5.28	1.75	5.14	9.38	10.01	4.42	4.11	5.95	5.54	7.96	3.74	11.36
2‐[2‐[2‐[2‐[2‐[2‐[2‐(2‐Hydroxyethoxy)ethoxy] ethoxy] ethoxy] ethoxy] ethoxy] ethoxy] ethanol	11.51	10.89	7.95	4.62	6.64	9.92	11.01	10.57	7.51	5.31	13.54	8.65	12.92	10.11
18,18′‐Bi‐1,4,7,10,13,16‐hexaoxacyclononadecane	1.34	0.99	0.50	0.59	0.66	1.29	2.03	0.54	0.31	1.18	2.21	1.09	1.19	1.08
1,4,7,10,13,16‐Hexaoxacyclooctadecane	13.30	8.25	2.59	4.58	3.80	10.73	10.50	10.85	5.63	4.46	6.93	13.54	8.52	1.76
3,6,9,12,15‐Pentaoxanonadecan‐1‐ol	0.01	0.39	0.12	0.02	0.02	0.20	1.31	0.01	0.06	0.02	0.62	0.00	0.15	0.00
2‐[2‐[2‐[2‐[2‐[2‐[2‐[2‐[2‐[2‐(2‐Methoxyethoxy)ethoxy] ethoxy]ethoxy]ethoxy]ethoxy] ethoxy]ethoxy]ethoxy]ethoxy]ethanol	0.35	1.46	0.07	0.11	0.80	1.93	0.09	1.11	0.58	0.05	0.72	0.87	0.08	1.52
3,3′‐Isopropylidenebis (1,5,8,11‐tetraoxacyclotridecane)	0.26	0.00	0.00	0.00	0.00	0.52	0.00	0.00	0.00	0.05	0.54	1.11	0.36	0.00
15‐Crown‐5	0.07	0.00	0.01	0.08	0.00	0.00	0.00	0.00	0.09	0.00	0.00	0.52	1.03	0.00
Benzene, 1‐methyl‐3‐(1‐methylethyl)—	0.55	0.00	0.00	0.00	0.00	0.00	0.00	0.00	0.00	0.00	0.00	0.00	0.00	0.00
2‐[2‐[2‐[2‐[2‐[2‐(2‐Methoxyethoxy)ethoxy] ethoxy] ethoxy] ethoxy] ethoxy] ethanol	0.00	0.00	3.14	0.03	0.00	0.00	0.00	0.00	0.00	3.84	0.00	0.00	0.00	0.00
2‐[2‐[2‐[2‐[2‐[2‐[2‐[2‐[2‐[2‐(2‐Hydroxyethoxy)ethoxy]ethoxy]ethoxy]ethoxy] ethoxy] ethoxy]ethoxy]ethoxy]ethoxy]ethanol	0.00	0.00	0.00	0.06	0.00	0.00	0.00	0.00	0.15	2.93	0.00	0.00	0.00	0.00
Ethyl 4‐(ethyloxy)‐2‐oxobut‐3‐enoate	0.00	0.00	0.00	0.00	0.00	0.20	0.00	0.00	0.00	0.00	0.00	0.00	0.00	0.00
2‐Methoxyethanol, TMS derivative	0.00	0.00	0.00	0.00	0.00	0.11	0.00	0.00	0.00	0.00	0.00	0.00	0.00	0.00
Isopropyl palmitate	0.00	0.00	0.00	0.00	0.00	0.00	0.24	0.00	0.00	0.00	0.00	0.00	0.00	0.00
3,5‐Octadien‐2‐one	0.00	0.00	0.00	0.00	0.00	0.00	0.46	0.00	0.00	0.00	0.00	0.00	0.00	0.00
Bicyclo[2.2.1]hept‐2‐ene, 2,7,7‐trimethyl—	0.00	0.00	0.00	0.00	0.00	0.00	0.00	0.89	0.00	2.39	0.00	0.00	0.00	0.00
2H‐Spiro[1‐benzofuran‐3,2′‐[1,3]dioxolane]‐5‐amine	0.00	0.00	0.00	0.00	0.00	0.00	0.00	0.66	0.72	0.00	0.49	0.54	1.11	0.58
4‐Amino‐5‐imidazole carboxamide, N,N,O‐ tris(trimethylsilyl)—	0.00	0.00	0.00	0.00	0.00	0.00	0.00	0.00	0.10	0.00	0.00	0.00	0.00	0.00
3,4‐Dihydroxymandelic acid, 4TMS derivative	0.00	0.00	0.00	0.00	0.00	0.00	0.00	0.00	0.27	0.17	0.14	0.23	0.00	0.22
Cyclohexene, 1‐methyl‐5‐(1‐methylethenyl)—	0.00	0.00	0.00	0.00	0.00	0.00	0.00	0.00	0.00	3.02	0.00	0.00	3.61	2.49
1(3H)‐Isobenzofuranone, 6‐(dimethylamino)‐3,3‐bis[4‐(dimethylamino)phenyl]—	0.00	0.00	0.00	0.00	0.00	0.00	0.00	0.00	0.00	0.09	0.06	0.00	0.00	0.09
Cyclopentene, 1‐ethenyl‐3‐methylene—	0.00	0.00	0.00	0.00	0.00	0.00	0.00	0.00	0.00	4.03	0.00	0.00	0.00	0.00
Cyclopropane, 1,1‐dimethyl‐2‐(3‐methyl‐1,3‐butadienyl)—	0.00	0.00	0.00	0.00	0.00	0.00	0.00	0.00	0.00	0.00	1.62	0.00	0.00	0.00
1,2,4‐Triazol‐4‐amine, 5‐ethyl‐3‐(3‐methyl‐5‐phenylpyrazol‐1‐yl)—	0.00	0.00	0.00	0.00	0.00	0.00	0.00	0.00	0.00	0.00	0.79	0.00	0.00	0.00
1,3,5‐Cycloheptatriene, 3,7,7‐trimethyl—	0.00	0.00	0.00	0.00	0.00	0.00	0.00	0.00	0.00	0.00	0.00	12.02	0.00	0.00
Library/ID	1.00	2.00	3.00	4.00	5.00	6.00	7.00	8.00	9.00	10.00	11.00	12.00	13.00	14.00
Benzene, 1,3‐dimethyl—	2.74	8.76	1.35	4.16	4.66	1.25	6.38	2.04	9.30	8.32	7.04	11.36	0.48	3.28

Different volatile compounds produced in different frying processes were also detected. 2‐Heptenal, (E)‐, 3,5‐ Octadien‐2‐ol, Benzene, 1,2‐dichloro‐, Furfural, 1‐Hexanol, 2‐ethyl‐, Benzaldehyde, Pentanoic acid and Phenylethyl Alcohol were detected in airfried chips, while Bicyclo[2.2.1]hept‐2‐ene, 2,7,7‐trimethyl‐, 2H‐Spiro[1‐ benzofuran‐3,2′‐[1,3]dioxolane]‐5‐amine, 4, Amino‐5‐imidazole carboxamide, N,N,O‐ tris(trimethylsilyl)‐, 3,4‐ Dihydroxymandelic acid, 4TMS derivative, Cyclohexene, 1‐methyl‐5‐(1‐methylethenyl)‐, 1(3H)‐ Isobenzofuranone, 6‐(dimethylamino)‐3,3‐bis[4‐(dimethylamino)phenyl]‐, Cyclopentene, 1‐ethenyl‐3‐methylene‐, Cyclopropane, 1,1‐dimethyl‐2‐(3‐methyl‐1,3‐butadienyl)‐, 1,2,4‐Triazol‐4‐amine, 5‐ethyl‐3‐(3‐ methyl‐5‐ phenylpyrazol‐1‐yl)‐ and 1,3,5‐Cycloheptatriene, 3,7,7‐trimethyl‐ were found in deep‐fried chips.

A total of 57 different volatile components were detected in chips produced with an air fryer, and 46 different volatile components were detected in chips produced with a deep fryer.

Pyrazines are typical products of the Maillard reaction (Mohamed et al. 2020). They are often associated with positive sensory perception, such as nutty, brown, roasted, and baked‐in fried potato chips, but they are also associated with negative sensory perception, such as raw and moldy (Agarwal et al. 2018). These negative flavor components were not found in gluten‐free bean chips. The substitution of beans for potatoes was positive in this sense.

In a study (Mohamed et al. 2020) looking at the aroma characteristics of different potato chips, the fact that one variety had 2.72 times more volatile compounds than the other variety was associated with the higher fat content as a reason for the higher amount of volatile compounds, while in gluten‐free bean chips, on the contrary, it was determined that those produced with an air fryer containing low fat had more volatile compounds. Different raw materials, frying conditions, and frying times may have caused this result.

In a study by Bredie et al. (1998) to investigate the flavors formed in corn flour during extrusion, increasing the product temperature, decreasing the moisture level, or extending the extrusion time increased the number and amount of compounds that are Maillard reaction products such as pyrazines, pyrroles, furans, and sulfur‐containing heterocycles. A significant increase in the amount of 2‐furfural, 2‐furanmethanol, and alkylpyrazine was determined in extrusion products at 180°C temperature and 14% moisture content. In our study, furfural was detected among these compounds, but other components were not found. Furfural was also found only in chips produced with an air fryer. With this result, it can be said that furfural is formed in chips that are not made in deep fat. In addition, He et al. (2021) emphasized that furfural and HMF formation occurs in a similar chemical pathway. According to our results, HMF was found more in samples fried with air‐fry. Thus, it was observed that furfural and HMF increased together by the literature.

The deep‐frying note originates from (E,E)‐2,4‐decadienal (Wagner and Grosch 1997). This is said to derive from the oxidation of linoleic acid, the most abundant fatty acid found in frying oils (Pangloli et al. 2002). When the amounts of (E,E)‐2,4‐decadienal in the samples were analyzed, it was found in both air fryer and deep fryer fried samples, while it was most abundant in chips produced in deep fat with a deep fryer, which means that chips have a frying note.

In the analysis of heat‐treated oils, hexanal, heptanal, octanal, nonanal, and 2‐decanal are identified as the specific malodors of saturated and unsaturated fatty acids. Hexanal is one of the most important secondary products formed during oxidation in foods containing linoleic acid or other 6‐carbon fatty acid oils and is used to monitor lipid oxidation. Volatile secondary oxidation products are important contributors to the odor and taste of oils and fried foods. The secondary oxidation products, polyunsaturated aldehydes such as 2,4‐decadienal, 2,4‐nonadienal, 2,4‐octadienal, 2‐heptanal, or 2‐octenal, are desirable aroma molecules that produce the characteristic fried aroma taste in oils, not as off‐flavors. However, saturated and unsaturated aldehydes such as hexanal, heptanal, octanal, nonanal, and 2‐decanal were found to have distinct off‐flavors in the analysis of heat‐treated oils. Fruity and plastic odors predominant in heat‐treated oils containing high oleic acid are primarily associated with heptanal, octanal, nonanal, and 2‐decanal (Akoh and Min 2002). Of the desirable compounds, all but 2,4‐octadienal were detected in the chips. 2,4‐decadienal was detected in both frying methods, while the others were only detected in chips produced by air fryer. Among the compounds responsible for the off flavor, only nonanal was found in chips produced with air fryer. From this point of view, it can be said that the frying process with air fryer is more favorable in terms of fried oil aroma profile. It is thought that the use of low oil amount is the reason for this flavor formation during heat treatment of cereals, involving thermally induced reactions such as the Maillard reaction and lipid degradation. Conditions such as temperature, water content, and residence time have been shown to have significant effects on the flavor profiles of products, and cooking temperature has been identified as the main influential factor in the formation of flavor compounds (Smith and Peterson 2020). Aroma compounds produced by the Maillard reaction or sugar degradation products include Strecker aldehydes, diketones, pyrazines, furans, acetic acid, and ethyl pyrrole. Compounds such as 2‐methyl butanal, 3‐methyl butanal, phenylacetaldehyde, and benzaldehyde are the Strecker aldehydes of the amino acids' isoleucine, leucine, and phenylalanine (Martin and Ames 2001a, 2001b). The major formation pathway appears to be oxidative deamination–decarboxylation of the corresponding amino acids via Strecker degradation (Sanches‐Silva et al. 2005). The fact that furfural, acetic acid, and benzaldehyde compounds are more abundant in chips produced with air fryer may indicate that the Maillard reaction occurs more. Long frying time may have caused this. As a matter of fact, the higher antioxidant activity and HMF amounts in these chips can also be explained by this.

**FIGURE 1:**
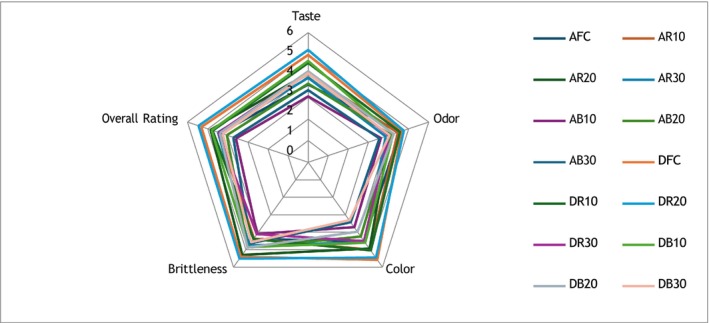
Spider graph of sensory evaluation of gluten‐free chips.

The data for the sensory evaluation of the chips is presented in Figure 1 as a spider plot on a scale of 0–7 points. The highest scoring samples in terms of taste were deep‐fried control chips, chips with 10% and 20% red bean flour substitution, and chips with 10% black bean substitution.

Although the hardness of the chips was higher in the hot air fryer, they had similar crispness scores in sensory analysis. When the general liking scores were analyzed, all results were above the average value of 3.50. It was found that the red bean flour substitutes received higher scores than the black ones. It is possible to say that the 20% bean flour substitutes were more liked because they received higher scores, although not statistically. The most disliked chips were the ones with 30% substitution. When all the criteria were considered, the deep‐fried control and the chips with 20% red bean flour substitution obtained the highest scores.

Yüksel et al. (2019) reported that gluten‐free chips produced by applying pre‐heat treatment received general liking scores out of 9 points: 4.70–5.85; taste/odor scores: 4.40–5.70 and hardness scores: 5.15–6.20. Yi et al. (2015) also produced potato chips with different frying methods and found that the scores of those deep‐fried in oil were higher in general taste. The results were found to be compatible with the data obtained in our study.

Although the chips produced with the air fryer were rich in terms of desired aroma components in frying oil and poor in terms of undesired ones, the taste scores were higher in chips produced in deep oil. For this reason, it was understood that only these compounds should not be considered when evaluating the taste of chips but should be evaluated with a holistic approach. In addition, oil is an important factor in the formation of taste, and the deep‐fried chips may have scored higher because of their higher oil content. In addition, the fact that chips produced in deep fat are crunchier may have supported this.

## 4 Conclusion

The results of this study provided detailed information on the production of gluten‐free chips using air fryer and deep fryer frying. Although it has shown that chips can be made with an air fryer, this technology, which is claimed to be healthier than deep frying, lags behind the deep fryer in terms of producing heat treatment contaminants (HMF). In fact, chips with more than 7.5 times less fat were produced, which was welcomed. These findings were important because they showed that consumers' focus on the amount of oil in this frying method alone may be an inadequate view. In terms of the quality of the chips, the results were in favor of air fryers. In terms of color, the air‐fried chips were more attractive, but with a higher degree of hardness. The color of the chips was closer to the control samples. However, the long cooking time and low pH were thought to increase the Maillard reaction, leading to an increase in the amount of HMF, while the total phenolic content and antioxidant activity were better preserved. Antioxidant activity results were not statistically very different. It is recommended to use different methods for this. Differences may not have been revealed by a single method. Except for 2,4‐octadienal, which is one of the desired compounds in fried foods, the others were detected in chips. 2,4‐decadienal was detected in both frying methods, while the other desirable compounds were only found in chips produced by air fryer. Among the compounds that contribute to the formation of off‐flavors, only nonanal was detected in chips produced with air fryer. These results indicate that the air fryer frying process is more favorable in terms of fried oil flavor profile, but this result is not reflected in the sensory analysis scores. This is thought to be due to the significant differences between the chips in terms of oil, texture, and other aroma information. In terms of the substituted gluten‐free bean flours, the chips produced with red bean flour rather than black bean flour received higher sensory scores. The chips fried in the deep fryer with 20% red bean flour substitute were even more liked than the control and had the highest scores. In addition to its rich nutritional composition, red bean flour was also preferred by consumers, which was a very positive result. Future studies are recommended to investigate the effect of Maillard reaction products and polycyclic aromatic hydrocarbons on the samples of different home frying methods.

## Conflicts of Interest

The author declares no conflicts of interest.

## Data Availability

The data that support the findings of this study are available from the corresponding author upon reasonable request.

## References

[fsn371065-bib-0001] Agarwal, D. , L. Mui , E. Aldridge , R. Mottram , J. McKinney , and I. D. Fisk . 2018. “The Impact of Nitrogen Gas Flushing on the Stability of Seasonings: Volatile Compounds and Sensory Perception of Cheese & Onion Seasoned Potato Crisps.” Food & Function 9, no. 9: 4730–4741. 30095140 10.1039/c8fo00817e PMC6148464

[fsn371065-bib-0002] Agarwal, S. , and E. S. Chauhan . 2022. “Nutraceutical Potential of Sweetened Chips Prepared by Gluten‐Free Composite Flour.” International Journal of Agriculture Environment and Biotechnology 15, no. 1: 141–145. 10.30954/0974-1712.01.2022.18.

[fsn371065-bib-0003] Akoh, C. C. , and D. B. Min . 2002. Food Lipids, Chemistry, Nutrition, and Biotechnology. Marcel Dekkel Inc.

[fsn371065-bib-0004] Altay, K. , A. R. Ergün , M. Şahin , and A. Deliboran . 2023. “Physico‐Mechanical, Nutritional, and Sensorial Properties of Gluten‐Free Chips With Almond Powder Dried Using Different Drying Methods.” Food Science and Engineering 4, no. 2: 159–347.

[fsn371065-bib-0005] AOAC . 2005. Official Methods of Analysis of the AOAC. 18th ed. AOAC.

[fsn371065-bib-0006] Barreto, N. M. , N. G. Pimenta , B. F. Braz , et al. 2021. “Organic Black Beans ( *Phaseolus vulgaris* L.) From Rio de Janeiro State, Brazil, Present More Phenolic Compounds and Better Nutritional Profile Than Nonorganic.” Food 10, no. 4: 900. 10.3390/foods10040900 PMC8073876 33921869

[fsn371065-bib-0007] Basaran, B. , and P. Sadighara . 2024. “The Level, Human Exposure, and Health Risk Assessment of Acrylamide in Chips and Breakfast Cereals: A Study From Türkiye.” Journal of Food Composition and Analysis 134: 106584.

[fsn371065-bib-0008] Bredie, W. L. P. , D. S. Mottram , and R. C. E. Guy . 1998. “Aroma Volatiles Generated During Extrusion Cooking of Maize Flour.” Journal of Agricultural and Food Chemistry 46: 1479–1487.

[fsn371065-bib-0009] Campos‐Vega, R. , R. Reynoso‐Camacho , G. Pedraza‐Aboytes , et al. 2009. “Chemical Composition and In Vitro Polysaccharide Fermentation of Different Beans ( *Phaseolus vulgaris* L.).” Journal of Food Science 74, no. 7: T59–T65. 19895501 10.1111/j.1750-3841.2009.01292.x

[fsn371065-bib-0010] Carbas, B. , N. Machado , D. Oppolzer , et al. 2020. “Nutrients, Antinutrients, Phenolic Composition, and Antioxidant Activity of Common Bean Cultivars and Their Potential for Food Applications.” Antioxidants 9, no. 2: 186. 32102193 10.3390/antiox9020186 PMC7070695

[fsn371065-bib-0011] Cengiz, S. , C. Kişmiroğlu , N. Cebi , J. Çatak , and M. Yaman . 2020. “Determination of the Most Potent Precursors of Advanced Glycation End Products (AGEs) in Chips, Crackers, and Breakfast Cereals by High Performance Liquid Chromatography (HPLC) Using Precolumn Derivatization With 4‐Nitro‐1, 2‐Phenlenediamine.” Microchemical Journal 158: 105170.

[fsn371065-bib-0012] Chávez‐Mendoza, C. , K. I. Hernández‐Figueroa , and E. Sánchez . 2018. “Antioxidant Capacity and Phytonutrient Content in the Seed Coat and Cotyledon of Common Beans ( *Phaseolus vulgaris* L.) From Various Regions in Mexico.” Antioxidants 8, no. 1: 5. 30585238 10.3390/antiox8010005 PMC6356214

[fsn371065-bib-0013] Comandini, P. , L. Cerretani , G. Blanda , A. Bendini , and T. G. Toschi . 2011. “Characterization of Potato Flavours: An Overview of Volatile Profiles and Analytical Procedures.” Potato V. Food 5: 1–14.

[fsn371065-bib-0014] D'Auria, M. , G. Mauriello , and G. L. Rana . 2004. “Volatile Organic Compounds From Saffron.” Flavour and Fragrance Journal 19, no. 1: 17–23. 10.1002/ffj.1266.

[fsn371065-bib-0015] de la Hera, E. , M. Talegón , P. Caballero , and M. Gómez . 2013. “Influence of Maize Flour Particle Size on Gluten‐Free Breadmaking.” Journal of the Science of Food and Agriculture 93, no. 4: 924–932. 22886488 10.1002/jsfa.5826

[fsn371065-bib-0016] Ding, P. , and Y. S. Ling . 2014. “Browning Assessment Methods and Polyphenol Oxidase in UV‐C Irradiated Berangan Banana Fruit.” International Food Research Journal 21, no. 4: 1667–1674.

[fsn371065-bib-0017] Ertekin Filiz, B. , and A. C. Seydim . 2018. “Kinetic Changes of Antioxidant Parameters, Ascorbic Acid Loss, and Hydroxymethyl Furfural Formation During Apple Chips Production.” Journal of Food Biochemistry 42, no. 6: e12676.

[fsn371065-bib-0018] Ertop, M. H. , K. Kutluk , K. Çoşkun , and S. Canlı . 2016. “A New Approach for Production of Chips With Food Industry Byproducts:Gluten Enriched Chips.” Akademik Gıda 14, no. 4: 398–406.

[fsn371065-bib-0019] Félix‐Medina, J. V. , J. Montes‐Ávila , R. Gutiérrez‐Dorado , et al. 2024. “Exploring Maillard Reaction Markers and Melanoidins to Investigate Toxicological and Antioxidant Profiles of Optimized Expanded Snacks From Corn/Common Bean Mixtures.” Journal of the Science of Food and Agriculture 104: 9035–9045. 38989963 10.1002/jsfa.13730

[fsn371065-bib-0020] Galgano, F. , N. Condelli , R. Tolve , et al. 2023. “Lentil Seed Coat as a Source of Phenolic Compounds: Influence of Geographical Origin and Genotype.” Food Measure 17: 1428–1437. 10.1007/s11694-022-01711-9.

[fsn371065-bib-0021] Gamez‐Meza, N. , J. A. Noriega‐Rodriguez , L. A. Medina‐Juarez , J. Ortega‐Garcia , R. Cazarez‐Casanova , and O. Angulo‐Guerrero . 1999. “Antioxidant Activity in Soybean Oil of Extracts From Thompson Grape Bagasse.” Journal of the American Oil Chemists' Society 76: 1445–1447. 10.1007/s11746-999-0182-4.

[fsn371065-bib-0022] Giovanelli, G. , L. Torri , and N. Sinelli . 2017. “Comparative Study of Physico‐Chemical and Sensory Characteristics of French Fries Prepared From Frozen Potatoes Using Different Cooking Systems.” European Food Research and Technology 243: 1619–1631. 10.1007/s00217-017-2870-x.

[fsn371065-bib-0023] Göncü, A. 2024. “The Effect of Using Sour Cherry ( *Prunus cerasus* L.) Puree in Tarhana Formulations on Nutritional Value and Functional Properties of Tarhana.” Food Science & Nutrition 12, no. 8: 5412–5425. 39139925 10.1002/fsn3.4191 PMC11317678

[fsn371065-bib-0024] Göncü, A. , and M. Hayta . 2018. “Rye and Oat Flour Enriched Baked Wheat Chips: Bioactive and Textural Properties.” Quality Assurance & Safety of Crops and Food 10, no. 1: 35–40.

[fsn371065-bib-0025] Gu, C. , L. Kong , X. Zhang , et al. 2024. “Effects of Black Bean Cell Wall Pectin by Exogenous Calcium Ions: Insight Into the Metabolomics, Physicochemical Properties and Anti‐Digestive Capacity.” International Journal of Biological Macromolecules 273: 133127. 38876245 10.1016/j.ijbiomac.2024.133127

[fsn371065-bib-0026] Guo, K. 2024. “Fabrication of a Molecular Imprinting Electrochemical Sensor for Detecting Acid Phosphatase in Black Bean Sprouts.” Food Measure 18: 640–646. 10.1007/s11694-023-02217-8.

[fsn371065-bib-0027] Halil, T. , C. E. Tamer , S. Suna , and A. Özkan Karabacak . 2020. “Investigations of Some Quality Parameters and Mathematical Modeling of Dried Functional Chips.” Heat and Mass Transfer 56: 1099–1115.

[fsn371065-bib-0028] Hayıt, F. , and H. Gül . 2017. “Çölyak ve Çölyak Hastaları Için Üretilen Ekmeklerin Kalite Özellikleri.” Journal of the Institute of Science and Technology 7, no. 1: 163–169.

[fsn371065-bib-0029] He, O. , Y. Zhang , P. Wang , et al. 2021. “Experimental and Kinetic Study on the Production of Furfural and HMF From Glucose.” Catalysts 11, no. 1: 11.

[fsn371065-bib-0030] Ho, C. T. , and Q. Chen . 1994. Lipids in Food Flavours. ACS Symposium Series. American Chemical Society.

[fsn371065-bib-0031] HunterLab . 1995. The Manual of Hunter‐Lab Mini Scan XE Colorimeter. HunterLab Cooperation.

[fsn371065-bib-0032] Kalani, P. , S. Jafarian , M. H. Azizi , G. H. Asadi , and L. R. Nasiraie . 2024. “Investigating the Effects of Fat and Sugar Reduction on the Rheological, Physicochemical, Microbial and Sensorial Characteristics of Gluten‐Free Functional Cup Cake.” Food Measure 18: 4930–4939.

[fsn371065-bib-0033] Kaplan, M. , F. Yüksel , and K. Karaman . 2021. “In Vitro Glycemic Index, Antioxidant Capacity and Some Physicochemical Characteristics of Deep‐Fried Sorghum Based Gluten Free Chips.” Journal of Food Science and Technology 58: 3725–3733. 34471296 10.1007/s13197-020-04830-7 PMC8357898

[fsn371065-bib-0034] Karakoç, F. B. 2021. “Menengiç, Susam ve Keten Tohumunun Bisküvi Formülasyonuna Ilavesinin Bisküvinin Kalitesi ve Raf Ömrü Üzerine Etkileri (Doctoral Dissertation, Necmettin Erbakan University).”

[fsn371065-bib-0035] Kayacier, A. , F. Yüksel , and S. Karaman . 2014. “Simplex Lattice Mixture Design Approach on Physicochemical and Sensory Properties of Wheat Chips Enriched With Different Legume Flours: An Optimization Study Based on Sensory Properties.” LWT‐Food Science and Technology 58, no. 2: 639–648.

[fsn371065-bib-0037] Le, D. T. , G. Kumar , G. Williamson , L. Devkota , and S. Dhital . 2024. “(Poly) Phenols and Dietary Fiber in Beans: Metabolism and Nutritional Impact in the Gastrointestinal Tract.” Food Hydrocolloids 156: 110350.

[fsn371065-bib-0038] Li, M. , J. Huang , Y. Chen , C. Liu , and X. Wu . 2024. “Protein From Red Adzuki Bean: Extraction Optimization, Glycosylation Modification and Physicochemical Properties of Glycation Products.” Food Measure 18: 4229–4245. 10.1007/s11694-024-02489-8.

[fsn371065-bib-0039] Lisińska, G. , and G. Gołubowska . 2005. “Structural Changes of Potato Tissue During French Fries Production.” Food Chemistry 93, no. 4: 681–687.

[fsn371065-bib-0040] Lozano‐Aguirre, M. G. , J. Rodríguez‐Miranda , R. N. Falfán‐Cortes , and B. Hernández‐Santos . 2023. “Physicochemical and Techno‐Functional Properties of Mixtures of Michigan Bean Protein Concentrate ( *Phaseolus vulgaris* L): Maltodextrin.” Food Measure 17: 1844–1851. 10.1007/s11694-022-01753-z.

[fsn371065-bib-0041] Mahmoud, R. M. , E. I. Yousif , M. G. Cadallah , and A. R. Alawneh . 2013. “Formulations and Quality Characterization of Gluten‐Free Egyptian Balady Flat Bread.” Annals of Agricultural Science 58: 19–25.

[fsn371065-bib-0042] Marta, H. , I. L. Anastasia , Y. Cahyana , F. Filianty , and D. Sondari . 2024. “Evaluation of Pasting and Functional Properties of Composite Flour From Indonesian Local Tubers and Its Application in Gluten‐Free Biscuits.” Food Measure 18: 6782–6792. 10.1007/s11694-024-02691-8.

[fsn371065-bib-0043] Martin, F. L. , and J. M. Ames . 2001a. “Comparison of Flavor Compounds of Potato Chips Fried in Palmolein and Silicone Fluid.” Journal of the American Oil Chemists' Society 78: 863–866.

[fsn371065-bib-0044] Martin, F. L. , and J. M. Ames . 2001b. “Formation of Strecker Aldehydes and Pyrazines in a Fried Potato Model System.” Journal of Agricultural and Food Chemistry 49, no. 8: 3885–3892. 11513684 10.1021/jf010310g

[fsn371065-bib-0045] Mazzola, A. M. , I. Zammarchi , M. C. Valerii , et al. 2024. “Gluten‐Free Diet and Other Celiac Disease Therapies: Current Understanding and Emerging Strategies.” Nutrients 16, no. 7: 1006. 10.3390/nu16071006. 38613039 PMC11013189

[fsn371065-bib-0046] Mesias, M. , C. Delgado‐Andrade , and F. J. Morales . 2019. “Risk/Benefit Evaluation of Traditional and Novel Formulations for Snacking: Acrylamide and Furfurals as Process Contaminants.” Journal of Food Composition and Analysis 79: 114–121.

[fsn371065-bib-0047] Mesías, M. , and F. J. Morales . 2017. “Effect of Different Flours on the Formation of Hydroxymethylfurfural, Furfural, and Dicarbonyl Compounds in Heated Glucose/Flour Systems.” Food 6, no. 2: 14. 10.3390/foods6020014 PMC5332907 28231092

[fsn371065-bib-0048] Mior Zakuan Azmi, M. , F. S. Taip , S. M. Mustapa Kamal , and N. L. Chin . 2019. “Effects of Temperature and Time on the Physical Characteristics of Moist Cakes Baked in Air Fryer.” Journal of Food Science and Technology 56: 4616–4624. 31686693 10.1007/s13197-019-03926-z PMC6801284

[fsn371065-bib-0049] Mohamed, M. , M. Ibrahim , H. Fadel , and S. Ghanem . 2020. “Comparative Study on the Volatile Compounds and Sensory Characteristics of Some Locally Produced Potato Chips.” Al‐Azhar Journal of Agricultural Research 45, no. 2: 33–48.

[fsn371065-bib-0077] Montaser, A. M. , A. S. Shahat , S. A. Al‐Shaimaa , and F. G. Salma . 2017. “Effects of Novel Antioxidants Composite on Oxidative Stability of Refined, Bleached, and Deodorized Palm Olein During Repeated Deep Frying of Potato Chips and Sensory Quality of Final Fried Food.” International Journal of Advanced Research 5, no. 7: 1791–1796.

[fsn371065-bib-0050] Morales, F. J. , and S. Jiménez‐Pérez . 2001. “Free Radical Scavenging Capacity of Maillard Reaction Products as Related to Colour and Fluorescence.” Food Chemistry 72, no. 1: 119–125.

[fsn371065-bib-0051] Moroni, A. V. , F. D. Bello , and E. K. Arendt . 2009. “Sourdough in Gluten‐Free Bread‐Making: an Ancient Technology to Solve a Novel Issue?” Food Microbiology 26: 676–684. 19747600 10.1016/j.fm.2009.07.001

[fsn371065-bib-0052] Olegario, L. S. , A. González‐Mohino , M. Estevéz , M. S. Madruga , and S. Ventanas . 2023. “Influence of Fat Reduction and Flavor Addition on the Temporal Sensory Profile in Potato Chips Using a Multiple‐Intake Approach.” Journal of the Science of Food and Agriculture 103, no. 10: 4934–4943. 36965131 10.1002/jsfa.12571

[fsn371065-bib-0053] Pangloli, P. , S. L. Melton , J. L. Collins , M. P. Penfield , and A. M. Saxton . 2002. “Flavor and Storage Stability of Potato Chips Fried in Cottonseed and Sunflower Oils and Palm Olein/Sunflower Oil Blends.” Journal of Food Science 67, no. 1: 97–103.

[fsn371065-bib-0054] Pedreschi, F. , A. Ferrera , A. Bunger , F. Alvarez , N. L. Huamán‐Castilla , and M. S. Mariotti‐Celis . 2021. “Ultrasonic‐Assisted Leaching of Glucose and Fructose as an Alternative Mitigation Technology of Acrylamide and 5‐Hydroxymethylfurfural in Potato Chips.” Innovative Food Science & Emerging Technologies 73: 102752.

[fsn371065-bib-0055] Qadri, T. , S. Z. Hussain , A. H. Rather , T. Amin , and B. Naseer . 2018. “Nutritional and Storage Stability of Wheat‐Based Crackers Incorporated With Brown Rice Flour and Carboxymethyl Cellulose (Cmc).” International Journal of Food Properties 21: 1117–1128.

[fsn371065-bib-0056] Rababah, T. M. , S. Brewer , W. Yang , et al. 2012. “Physicochemical Properties of Fortified Corn Chips With Broad Bean Flour, Chickpea Flour or Isolated Soy Protein.” Journal of Food Quality 35, no. 3: 200–206.

[fsn371065-bib-0057] Rocchetti, G. , G. Giuberti , and L. Lucini . 2018. “Gluten‐Free Cereal‐Based Food Products: The Potential of Metabolomics to Investigate Changes in Phenolics Profile and Their in Vitro Bioaccessibility.” Current Opinion in Food Science 22: 1–8.

[fsn371065-bib-0058] Sabença, C. , M. Ribeiro , T. Sousa , P. Poeta , A. S. Bagulho , and G. Igrejas . 2021. “Wheat/Gluten‐Related Disorders and Gluten‐Free Diet Misconceptions: A Review.” Food 10: 1765. 10.3390/foods10081765 PMC8391170 34441542

[fsn371065-bib-0059] Sanches‐Silva, A. , J. Lopez‐Hernández , and P. Paseiro‐Losada . 2005. “Profiling Flavour Compounds of Potato Crisps During Storage Using Solid‐Phase Microextraction.” Journal of Chromatography A 1064, no. 2: 239–245. 15739892 10.1016/j.chroma.2004.05.108

[fsn371065-bib-0060] Sancho, R. A. S. , V. Pavan , and G. M. Pastore . 2015. “Effect of in Vitro Digestion on Bioactive Compounds and Antioxidant Activity of Common Bean Seed Coats.” Food Research International 76: 74–78.

[fsn371065-bib-0061] Smith, K. , and D. G. Peterson . 2020. “Identification of Aroma Differences in Refined and Whole Grain Extruded Maize Puffs.” Molecules 25, no. 9: 2261. 32403322 10.3390/molecules25092261 PMC7249081

[fsn371065-bib-0062] Taylord, A. J. , and R. S. T. Linforth . 2010. Food Flavour Technology. Wiley‐Blackwell.

[fsn371065-bib-0063] Wagner, R. , and W. Grosch . 1997. “Evaluation of Potent Odorants of French Fries.” LWT‐Food Science and Technology 30, no. 2: 164–169.

[fsn371065-bib-0064] Wesley, S. D. , B. H. M. André , and M. T. P. S. Clerici . 2021. “Gluten‐Free Rice & Bean Biscuit: Characterization of a New Food Product.” Heliyon 7, no. 1: e05956. 33521353 10.1016/j.heliyon.2021.e05956 PMC7820923

[fsn371065-bib-0065] Xiong, K. , M. M. Li , Y. Q. Chen , Y. M. Hu , and W. Jin . 2024. “Formation and Reductionof Toxic Compounds Derived From the Maillard Reaction During the Thermal Processing of Different Food Matrices.” Journal of Food Protection 87, no. 9: 100338. 39103091 10.1016/j.jfp.2024.100338

[fsn371065-bib-0066] Yakıcı, T. 2012. Farklı Reçetelerle Üretilen Bisküvilerde Aroma ve Akrilamid Oluşumu. Doktora Tezi. Namık Kemal Üniversitesi, Fen Bilimleri Enstitüsü.

[fsn371065-bib-0067] Yang, H. , L. Li , Y. Yin , et al. 2019. “Effect of Ground Ginger on Dough and Biscuit Characteristics and Acrylamide Content.” Food Science and Biotechnology 28: 1359–1366. 10.1007/s10068-019-00592-x. 31695934 PMC6811461

[fsn371065-bib-0068] Yi, H. , K. T. Hwang , H. Choi , and H. T. Lim . 2015. “Physicochemical and Organoleptic Characteristics of Deep‐Fat Fried and Microwaved Potato Chips.” Journal of Korean Society for Applied Biological Chemistry 58: 735–740.

[fsn371065-bib-0069] Yıldırım Vardin, A. 2024. “The Effects of Microwave Assisted Baking on Maillard Reaction Products in Gluten Free Biscuits Produced With Different Formulations.” PhD. Thesis. Aydın Adnan Menderes University.

[fsn371065-bib-0070] Yuksel, F. 2017. “Effect of Powder of Macaroni Boiling Water (By‐Product) on Textural, Oil Uptake, Physico‐Chemical, Sensory and Morphological Properties of Fried Wheat Chips.” Food Measure 11: 290–298. 10.1007/s11694-016-9396-y.

[fsn371065-bib-0071] Yuksel, F. , S. Karaman , M. Gurbuz , et al. 2017. “Production of Deep‐Fried Corn Chips Using Stale Bread Powder: Effect of Frying Time, Temperature and Concentration.” LWT‐Food Science and Technology 83: 235–242.

[fsn371065-bib-0072] Yuksel, F. , and A. Kayacier . 2016. “Utilization of Stale Bread in Fried Wheat Chips: Response Surface Methodology Study for the Characterization of Textural, Morphologic, Sensory, Some Physicochemical and Chemical Properties of Wheat Chips.” LWT‐Food Science and Technology 67: 89–98.

[fsn371065-bib-0073] Yüksel, F. 2014. “Utulization of Stale Bread in Fried Wheat and Corn Chips (PhD Thesis. Erciyes University).”

[fsn371065-bib-0074] Yüksel, F. , B. Yavuz , and A. Durmaz . 2019. “Determination of Oil Uptake Capacities and Some Physiochemical Analyses With Sensory Properties of Fried Gluten Free Chips After That Subjected to Pre‐Drying Process at Different Temperatures and Times.” Turkish Journal of Agriculture‐Food Science and Technology 7, no. 3: 384–389.

[fsn371065-bib-0075] Zhang, Z. , Y. Chen , P. Deng , et al. 2024. “Research Progress on Generation, Detection and Inhibition of Multiple Hazards‐Acrylamide, 5‐Hydroxymethylfurfural, Advanced Glycation End Products, Methylimidazole‐In Baked Goods.” Food Chemistry 431: 137152. 37603996 10.1016/j.foodchem.2023.137152

[fsn371065-bib-0076] Žilić, S. , V. Hadži‐Tašković Šukalović , M. Milašinović , D. Ignjatović‐Micić , M. Maksimović , and V. Semenčenko . 2010. “Effect of Micronisation on the Composition and Properties of the Flour From White, Yellow and Red Maize.” Food Technology and Biotechnology 48, no. 2: 198–206.

